# Risk factors for arbovirus infections in a low-income community of Rio de Janeiro, Brazil, 2015-2016

**DOI:** 10.1371/journal.pone.0198357

**Published:** 2018-06-07

**Authors:** Nádia Cristina Pinheiro Rodrigues, Regina Paiva Daumas, Andrea Sobral de Almeida, Reinaldo Souza dos Santos, Isabella Koster, Pedro Pinheiro Rodrigues, Marcelly de Freitas Gomes, Auriane de Fátima Macedo, Alyssa Gerardi, Iúri da Costa Leite

**Affiliations:** 1 National School of Public Health Sergio Arouca, Oswaldo Cruz Foundation, Rio de Janeiro, Brazil; 2 School of Medical Sciences, Rio de Janeiro State University, Rio de Janeiro, Brazil; 3 Universidade Federal Fluminense, Niterói, Brazil; 4 Georgetown University, Washington, DC, United States of America; Columbia University, UNITED STATES

## Abstract

**Background:**

Dengue epidemics have occurred in the city of Rio de Janeiro (Brazil) since 1986. In the year 2015, Zika and chikungunya viruses were introduced in the city, causing sequential and simultaneous epidemics. Poor socioeconomic conditions have been suggested as contributing factors of arboviral infection.

**Objective:**

To describe the spatial distribution of human cases of symptomatic arboviral infections and to identify risk factors for infection in a poor community of Rio de Janeiro in the years 2015 and 2016.

**Methods:**

We built thematic maps of incidence rates for 78 micro-areas in the Manguinhos neighborhood. The micro-areas congregate about 600 inhabitants. Simple and multiple multilevel logistic regression models were used to evaluate the association between the incidence of arboviral diseases and socio-demographic factors at both the individual and micro-area levels.

**Results:**

From 2015 to 2016, 370 human cases of arbovirus infection were reported in the Manguinhos community: 123 in 2015 and 247 in 2016. There was a significant difference in the risk of arbovirus diseases among different micro-areas, but this was not explained by water and sanitation indicators. The cumulative incidence rate was 849/100,000 in two years. The incidence was greater in those individuals with familiar vulnerability (1,156/100,000 vs. 794/100,000). The multilevel adjusted model showed that the odds of acquiring an arbovirus infection was 55% greater in those with familiar vulnerability.

**Conclusion:**

Arbovirus infections cause a high burden of disease in Brazilian urban centers. Our results suggest that even in poor neighborhoods, there is a high spatial variability in the risk of acquiring an arbovirus infection. The conditions that favor vector proliferation and infection by arboviruses are complex and involve both individual and environmental characteristics that vary from place to place. To reduce the burden of arboviral diseases, continued public health policies and basic services should be provided to the communities at risk that consider specific local needs.

## Introduction

Dengue, Zika and chikungunya are acute viral diseases caused by arthropod-borne viruses (arboviruses). Dengue is the most frequent disease transmitted by mosquitoes in the world, with an incidence that has dramatically increased in the last five decades [1]. It is endemic in Brazil, where all four dengue virus (DENV) serotypes circulate, causing large epidemics in urban areas [2, 3]. Zika and chikungunya were recently introduced to the Americas and have spread rapidly in Brazil [4, 5].

Dengue and chikungunya may be clinically indistinguishable in the first days of disease as both typically present with high grade fevers, body aches, and a variety of other general symptoms such as headache and rash. Zika usually presents as a pruritic rash with mild to moderate arthralgia and a low-grade fever or no fever at all. It may also be misdiagnosed as dengue, especially in areas where it is endemic [6]. Although Zika virus (ZIKV) causes the mildest symptoms of the three diseases, this infection is especially feared by pregnant women since it may cause congenital malformations, like microcephaly [7, 8].

*Aedes aegypti*, a highly anthropophilic mosquito, is the main vector of these diseases in the Americas [9]. Unplanned urban growth, increasing mobility and international trade have contributed to its spread in tropical urban areas all over the world [10]. The process of urbanization harms the integrity of native habitats of many species. As men become the most available and stable source of blood, these insects live and procreate in close proximity to them, which promotes their propagation to other environments. In addition, with human mobility, these evolved species spread more and more [11].

The city of Rio de Janeiro, the second largest urban center in Brazil, has had five major dengue epidemics since 1986 [12]. In 2015, Zika and chikungunya viruses were isolated for the first time in the city [13]. Their arrival in a city already infested by *Aedes aegypti* mosquitoes with a susceptible human population caused sequential epidemics in the years 2015 and 2016. The absence of vaccines to prevent chikungunya and Zika, coupled with the fact that all three diseases share a common vector, highlights the importance of vector management strategies to achieve their control [10].

Planning and evaluation of vector management strategies depend on identifying high risk areas and predictors of increased risk. The interaction of social, environmental, ecological and climatic factors determines the distribution of mosquitoes as well as the human exposure to vector-borne-diseases [14]. Housing type, population density, population flow and host factors such as age, sex, comorbidities and immunity are also important factors reported in literature as predictors [15–17].

Although Brazilian health managers have been trying to reduce dengue incidence the past few decades, they have not yet achieved satisfactory results. The growth of slums and irregular constructions in overcrowded urban centers and associated poor sanitation and unsatisfactory garbage collection favor the reproduction of *Aedes aegypti*, making it difficult to control dengue and other *Aedes* transmitted diseases as a result [1, 18]. Poor urban areas may be especially prone to *Aedes aegypti* infestation because the lack of water supply and garbage collection can result in breeding sites for these mosquitoes [1, 19].

The present study is focused on describing the spatial distribution pattern of symptomatic arbovirus infections in Manguinhos, a low-income neighborhood of Rio de Janeiro (Brazil), and analyzing their relationship with socio-demographic factors at both the individual and aggregate levels.

## Methods

**Fig 1:**
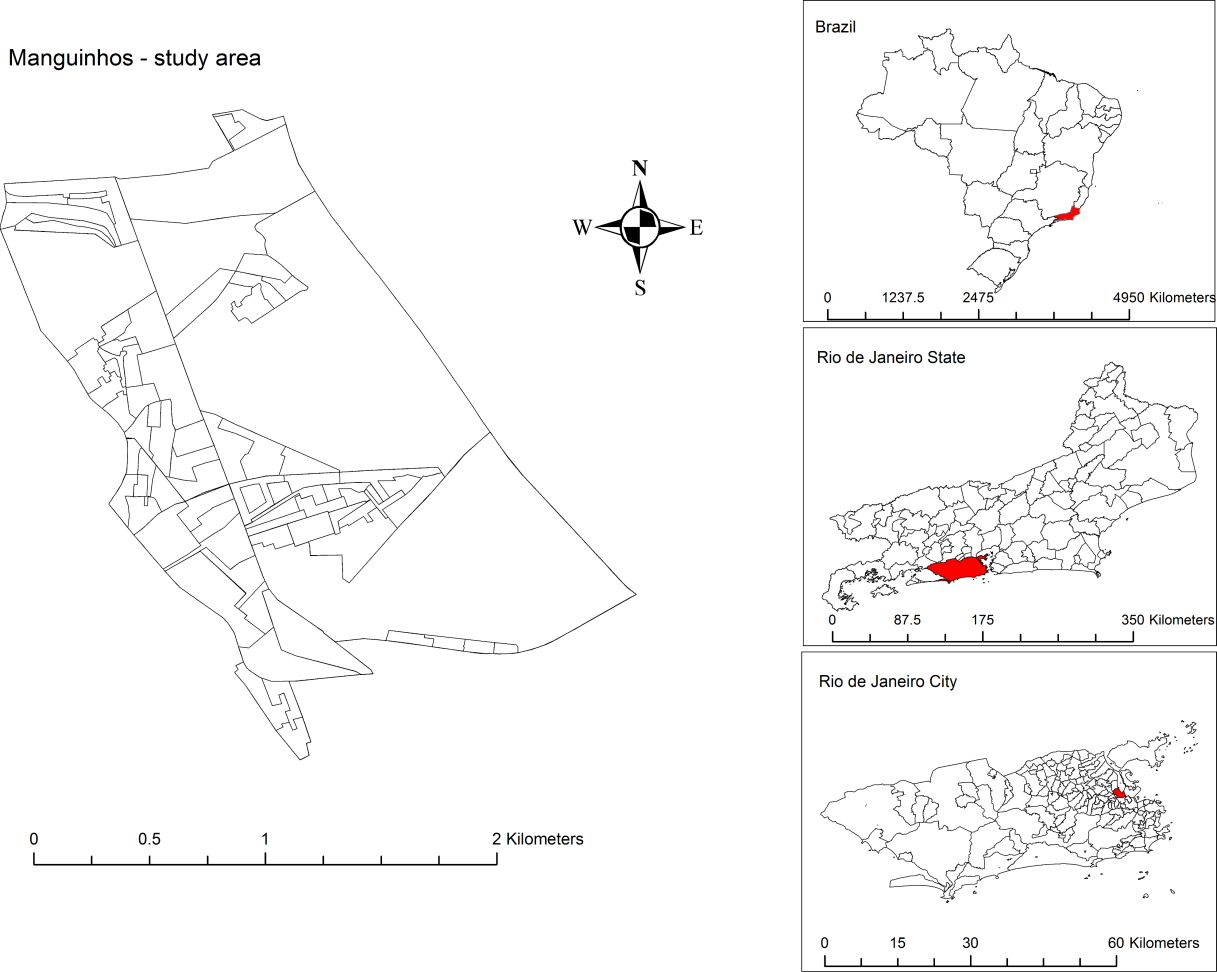
Location of the study area (Manguinhos community) in Brazil according to its state and city.

This is a cross sectional study (2015–2016) using surveillance data of the primary health care services that assist the population of Manguinhos, a low-income neighborhood of the city of Rio de Janeiro city (Brazil) (Fig 1). Manguinhos is a neighborhood of 261.84 hectares located in the north zone of Rio de Janeiro city. It has the fourth lowest family income per capita among the 117 neighborhoods and neighborhood groups in the city and approximately 75% of its population lives in slums (IBGE, Censo 2010). The average monthly familiar income in Rio de Janeiro City in 2010 was USD 629.18, while in Manguinhos it was USD 308.87 [20–22].

The area is completely covered by Brazil’s Family Health Strategy, which delivers community-based primary care through the country’s Universal Health System. In Manguinhos, each of the 13 family health care teams assists one meso-area with approximately 3500 people. Each meso-area is further divided into six micro-areas that are fairly homogeneous in relation to socio-economic and environmental factors. The micro-areas congregate about 600 inhabitants [23]. A community health agent is assigned to each micro-area and is responsible for registering and following-up with the families. By January 2016, there were 43,282 people registered in the ascribed area.

We estimated incidence data for both the meso- (n = 13) and micro-areas (n = 78).We did not estimate disease rates in four non-residential areas.

Data were collected from notification forms of suspected human cases of dengue, Zika and chikungunya that health professionals reported to the surveillance system. We worked with all suspected cases of arboviral diseases as a group because we were interested in the risk of transmission of any mosquito-borne disease in the area. However, the infections were frequently confounded and not all cases were laboratory-confirmed. Zika and chikungunya were particularly unrecognized in the beginning and were sometimes misdiagnosed as dengue. Laboratory confirmation was not available for all suspected cases of Zika and was restricted to pregnant women since there was no valid serological test at the time. Blood collection was performed in the local health units. PCR tests for Zika and enzyme immunoassays to detect IgM for chikungunya and dengue were performed by the public health central state laboratory (LACEN-RJ) according to physicians’ requests. In order to provide information on the circulating arboviruses, the rate of laboratory confirmation for each disease was calculated.

A geo-referenced mesh of meso-areas and micro-areas provided by the health care service was used, and individual data on socio-demographic and housing features were collected from the local database called the Primary Care Information System (SIAB). Data on familiar vulnerability were also available in the database as it was previously estimated by local health managers. SIAB housing and socio-demographic data are collected at home visits by community health workers when they first register families who live in the area. These data are regularly revised and updated. Family Risk Scale data were collected in the same way.

### Statistical analysis

We carried out bivariate analyses to evaluate the association between individual characteristics and the risk of acquiring an arbovirus infection in the years 2015 and 2016. We evaluated age (in years), sex (male, female), race (white, nonwhite), monthly family income (low: ≤ USD 319.15), moderate: > USD 319.15), receiving government aid (yes/no; financial support from government programs), possession of private health insurance (yes/no) and family vulnerability (yes/no; classified according to the adapted Family Risk Scale) [24]. The Family Risk Scale includes information about age, schooling, habitation, employment, drug use, nutrition and morbidity, which then provides a measurement based on indicators of risk and vulnerabilities. In addition to biological aspects of the population obtained by the health care services, the scale incorporates socioeconomic, cultural and environmental factors that contribute to greater knowledge of the health determinants. A family with a score greater than or equal to 5 is considered vulnerable [24] (Table 1).

**Table 1:** Distribution of the scores of the adapted Family Risk Scale.

Factor (level)	Score
**Illiterate^1^ (individual)**	1
**Lack of health insurance (individual)**	1
**Home built with recycled materials (family)**	3
**Agglomeration (family)**	
**High**	3
**Moderate**	2
**Low**	0
**No garbage collection (family)**	3
**Untreated water (family)**	3
**Open sewage (family)**	3
**Person who lives alone (individual)**	1
**Unemployed (individual)**	2
**Drug user (individual)**	2
**Alcoholic (individual)**	2
**Handicapped person (individual)**	3
**Diabetic (individual)**	1
**Pregnant (individual)**	1
**Hypertensive (individual)**	1
**Monthly income lower than 319.15USD (family)**	1
**Family benefited with government aid (family)**	1
**Age lower than two years old (individual)**	1
**Age higher than 60 years old (individual)**	1

^1^For those older than six years old.

Unadjusted odds ratios were estimated in simple multilevel logistic models for both individual and aggregate variables at the family level. Every variable that showed a p-value lower than 0.20 in the simple analysis was included in the multiple regression model. The individual variables investigated were: gender, race, age and private health insurance, while the others were aggregate variables. As socioeconomic factors can vary between groups and according to location, random effects of the micro-areas and meso-areas were assessed. Using point data, we plotted the distribution of arboviral disease human cases in the Manguinhos community map. We built Kernel Density maps to estimate the predicted density value. We used planar distances between the features and defined square kilometers as the output area density units. The search radius (bandwidth) was computed to each dataset using a spatial variant of Silverman's Rule of Thumb [25]. Using area data, we calculated the rate of arbovirus infection by micro-area. We used the Getis-Ord Gi statistic, with a fixed Euclidean distance band (Distance Band = 100m) to identify statistically significant hot spots and cold spots. Areas with non-residential buildings such as the Fundação Oswaldo Cruz (Institution of Science and Technology in Health), a post office, an oil refinery (recently reactivated in 2015) and a shelter for the elderly surrounded by an extensive uninhabited area were excluded to prevent bias in this analysis.

We used tables and maps to present the results. All analyses were performed using ArcGis (version 10.4), MLwiN (version 3.0) and R-Project (version 3.4.1) software.

This study was approved by the Ethics and Research Committee of Escola Nacional de Saúde Pública Sérgio Arouca / Fundação Oswaldo Cruz—number 1,545,850. The Ethics Committee waived the requirement for informed consent as it would not be possible to access each patient recorded. All data were fully anonymized before the analysis of the research team.

## Results

From 2015 to 2016, there were 370 human cases of arbovirus infection in the Manguinhos community: 123 in 2015 and 247 in 2016. The incidence rate ranged from 284/100,000 in 2015 to 571/100,000 in 2016, with a cumulative incidence rate of 849/100,000 in the two-year period. Among these cases, 21% were reported as Zika, 31% as chikungunya and 48% as dengue. All of the chikungunya cases and the majority of the Zika cases reported were in 2016. The average age of the population in the community and the average age of those with arboviral infections were 33 (Standard Deviation (SD) = 20.8) and 35 (SD = 19.9) years old, respectively. Table 2 presents the accumulated incidence rate of arboviral diseases in the 2015 to 2016 periods according to sex, age, color/race, family income, familiar vulnerability, government aid, possession of private health insurance and households with the following: a piped water sewage system and garbage collection. Incidence was greater among females (1,042/100,000 vs. 628/100,000) and those with familiar vulnerability (1,156/100,000 vs. 794/100,000). No other factor was significantly associated with contracting an arboviral disease. The multilevel adjusted model showed that the odds of arbovirus infection in males was 42% lower than in females (OR = 0.58) and those classified with familiar vulnerability had 55% greater odds than those who were not.

**Table 2:** Unadjusted and adjusted odds ratios and 95 confidence intervals for a set of factors associated with arbovirus infection, 2015–2016.

Factors	Groups	Total	Cumulative incidence	Unadjusted	P-v	Adjusted	P-v
			n (rate/100,000)	OR (95% CI)		OR (95% CI)	
**Sex**	Female	23,694	247 (1,042)	1.00	NA	1.00	NA
	Male	19,588	123 (628)	0.60 (0.48–0.75)	<0.01	0.60 (0.46–0.75)	<0.01
**Age**				1.01 (0.98–1.04)	0.51	NA	NA
**Race**	White	18,053	164 (908)	1.00	NA	NA	NA
	Nonwhite	23,635	192 (812)	0.96 (0.77–1.20)	0.74	NA	NA
**Familiar income**	Low	22896	201 (877)	1.08 (0.79–1.50)	0.62	NA	NA
	Moderate	6,057	59 (974)	1.00	NA	NA	NA
**Familiar vulnerability**	Yes	10,034	106 (1,056)	1.50 (1.18–1.92)	<0.01	1.50 (1.17–1.91)	<0.01
	No	33,248	264 (794)	1.00	NA	1.00	NA
**Government aid**	Yes	4,524	42 (928)	1.11 (0.78–1.57)	0.58	NA	NA
	No	38,758	328 (846)	1.00	NA	NA	NA
**Private health insurance**	Yes	4,176	40 (958)	1.12 (0.78–1.60)	0.53	NA	NA
	No	39,106	330 (844)	1.00	NA	NA	NA
**Piped water system**	Yes	41911	361(861)	NA	NA	NA	NA
	No	124	0(0)	NA	NA	NA	NA
**Sewerage system**	Yes	40921	351(857)	1.00	NA	NA	NA
	No	1130	10(885)	1.08 (0.50–2.31)	0.85	NA	NA
**Garbage collection**	Yes	39513	341(863)	1.00	NA	NA	NA
	No	2364	20(846)	1.10(0.65–1.88)	0.72	NA	NA

P-v = p- value; OR = odds ratio; NA = not applicable

Analyzing the random effects, no significant difference between the coefficients, standard deviations and p-values of unadjusted and adjusted models were detected (Table 3). The intra-class correlation coefficient of the models indicates that approximately 6% of arbovirus infection variance is associated with the variability of the meso-areas (p-value < 0.07), while around 12% of arbovirus infection variance is associated with the variability of the micro-areas (p-value < 0.01) (Table 3).

**Table 3:** Random effects, standard errors and intra-class correlation coefficients (ICC) at meso and micro-areas of a low-income community of Rio de Janeiro city.

	Unadjusted Model			
Random effects	Coefficients	SE	p-value	ICC %
Meso $\left(\sigma_{k}^{2}\right)$	0.22	0.12	0.07	5.8
Micro $\left(\sigma_{jk}^{2}\right)$	0.23	0.08	0.01	11.9
	Adjusted Model			
Meso $\left(\sigma_{k}^{2}\right)$	0.24	0.13	0.07	6.4
Micro $\left(\sigma_{jk}^{2}\right)$	0.23	0.09	0.01	12.4

SE = Standard Deviation; ICC = Intra-class Correlation Coefficient

While the expected rate (per 100,000) of acquiring an arbovirus infection ranges from 545 to 1401 for those with familiar vulnerability, the expected range is lower for those without it (364 to 940) (Table 4). However, we found that this rate also varies according to the effect of the micro-area. A person from a vulnerable family who lives in a micro-area with a below-average risk of infection has a low risk of being infected (545/100,000). On the other hand, a person who from a non-vulnerable family who lives in a micro-area with an above-average risk of infection also has a high risk of being infected (940/100,000). A person has the highest risk of infection (1401/100,000) if he belongs to a vulnerable family and lives in a micro-area with an above-average risk of infection (Table 4).

**Table 4:** Expected number (per 100,000) of acquiring arbovirus infection according to sex, social vulnerability and levels of micro-area effects.

Factors	Micro-area effect		
	Below Average	Average	Above Average
Social Vulnerability			
Yes	545	874	1401
No	364	586	940
Sex			
Female	504	810	1298
Male	302	486	780

While the expected rate (per 100,000) of acquiring an arbovirus infection ranges from 504 to 1298 for females, the expected range is lower for males (302 to 780) (Table 4). Again, this number also varies according to the effect of the micro-area. Women who live in a micro-area with a below-average risk of infection have a low risk of being infected (504/100,000) and men who live in a micro-area with an above-average risk of infection have higher risk of being infected (780/100,000) (Table 4).

**Fig 2:**
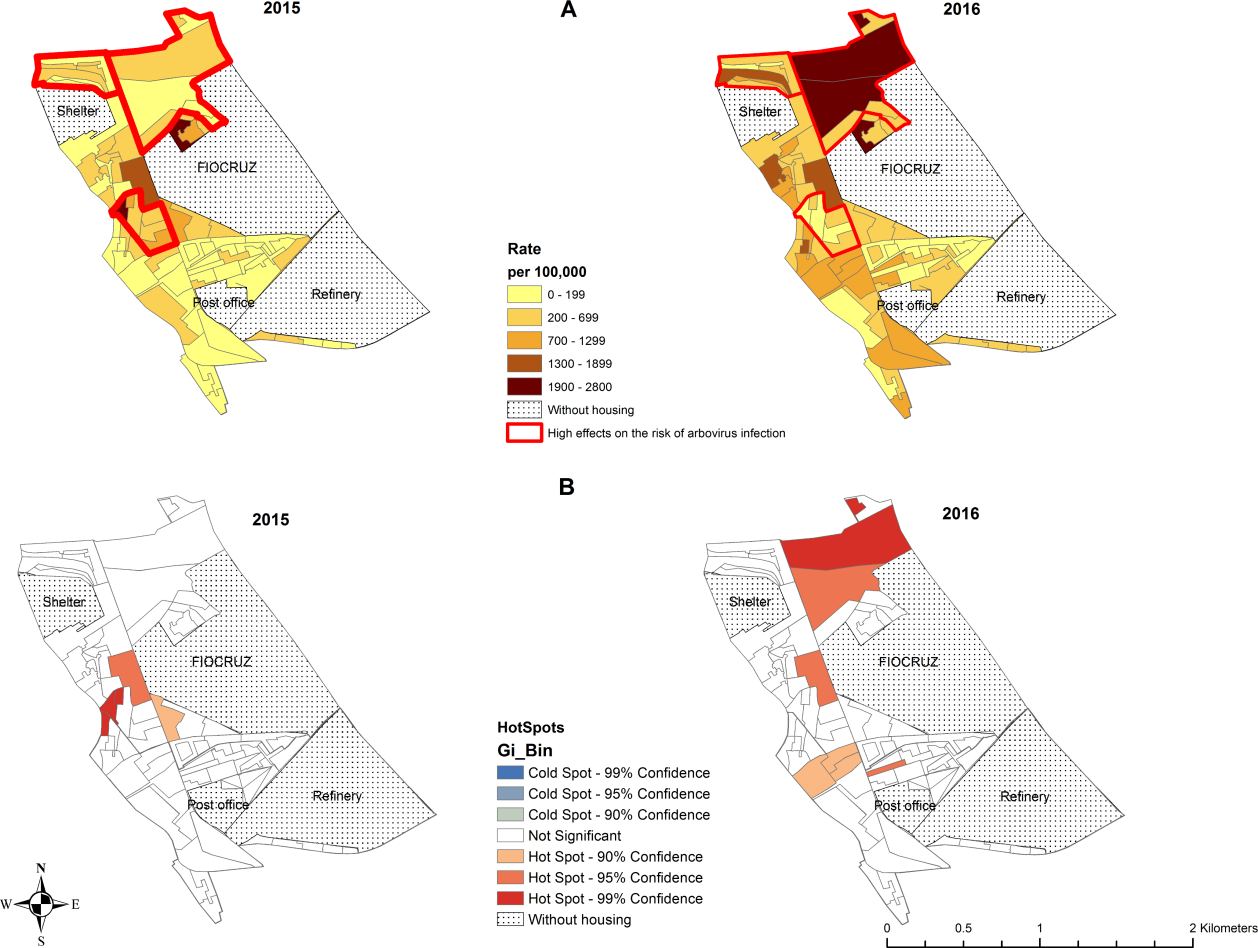
Distribution of arbovirus infection rate (dengue, Zika and chikunguya) in the micro-areas of a low-income community, Rio de Janeiro (Brazil). FIOCRUZ = Fundação Oswaldo Cruz (institution of science and technology in health); Refinery: oil refinery; Shelter = Shelter for the elderly.

Analyzing the random effects of micro-areas, we can see that three micro-areas demonstrate significantly high effects on the risk of arbovirus infection (Fig 2A). These micro-areas present rates higher than 1,300 per 100,000.

**Fig 3:**
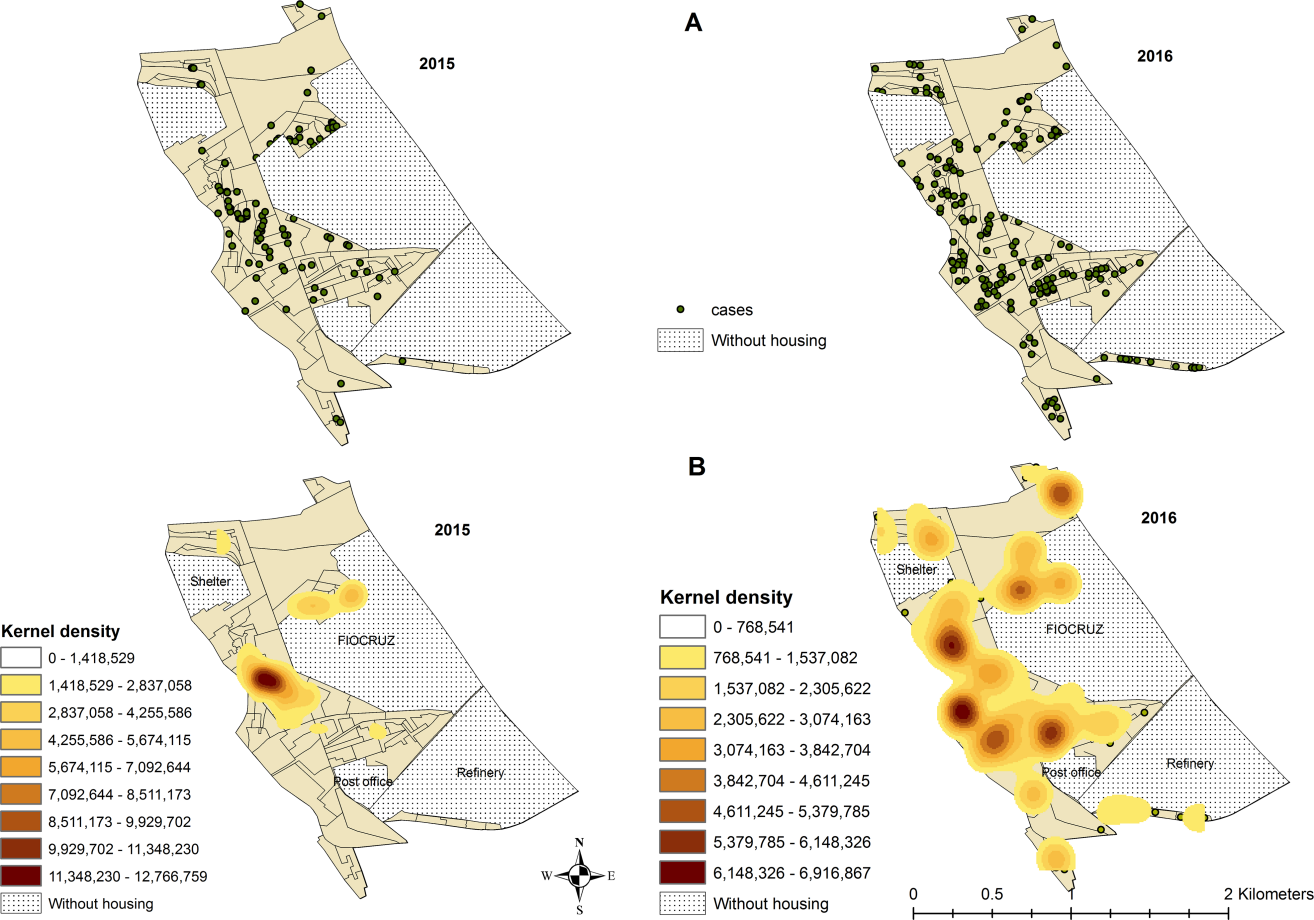
Distribution of the human cases of arbovirus infection (dengue, Zika and chikunguya) in the micro-areas of a low-income community of Rio de Janeiro (Brazil). FIOCRUZ = Fundação Oswaldo Cruz (institution of science and technology in health); Refinery: oil refinery; Shelter = Shelter for the elderly.

In observing the spatial distribution of human cases in the years 2015 and 2016 (Fig 3A), the micro-areas with the highest number of cases in 2015 and 2016 were those in the West, the North and between Fundação Oswaldo Cruz (FIOCRUZ) and the oil refinery. In 2016, we observed new cases in the South. The Kernel map (Fig 3B) shows that in 2015, the main cluster of cases was located to the West of FIOCRUZ. Another cluster with lower density could also be observed to the North of FIOCRUZ. In 2016, there were several clusters of similar density spread out along the West, Northwest, Southwest and North of FIOCRUZ.

The micro-areas with the highest incidence rates in 2015 were located to the West and to the North of FIOCRUZ. In 2016, micro-areas in the West, North and Northwest showed the highest rates (Fig 2A).

The hot spots are micro-areas with a high incidence surrounded by other areas that also have a high incidence (Fig 3B). In 2015, significant hot spots were observed in micro-areas in the West. In 2016, in addition to the micro-areas in the West, hot spots were located in the North and between FIOCRUZ and the refinery (Fig 2B). Confirmatory laboratory tests were performed in 33% of the all suspected human cases. The percentage of suspected cases tested varied by disease: 48% of chikungunya, 33% of dengue and 6% of Zika suspected cases. Test results were positive in 73% of chikungunya and 20% of Zika infection suspected cases who underwent tests. All laboratory tests for dengue were negative.

## Discussion

We studied the distribution and risk factors for diseases transmitted by *Aedes* mosquitoes among people living in a poor community in Rio de Janeiro during a period when three arboviruses circulated. We found significant differences in the risk of arbovirus diseases among different micro-areas that were not explained by the traditional water and sanitation indicators. From 2015 to 2016, incidence rates of arboviral diseases increased significantly in Manguinhos, as also occurred in the entire city. The introduction of Zika in 2015 and chikungunya in 2016 in this area may explain this growth. In the city of Rio de Janeiro, an epidemic of an unknown exanthematic disease, later recognized as Zika, was first reported in April 2015. By November 2015, it became mandatory to report Zika cases. A total of 7,226 Zika cases were reported in 2015 and 31,966 cases in 2016. By the end of 2015, the first few autochthonous chikungunya cases were also reported, evolving to an epidemic in the next year, with 14,202 chikungunya cases and 20 deaths reported in 2016 [26]. Many dengue cases were also reported both in 2015 (18,070) and 2016 (25,838) in the city of Rio de Janeiro. However, it is likely that most suspected cases of Zika and chikungunya were misdiagnosed as dengue, as suggested by the high percentage of laboratory-negative dengue suspected cases and the time-relation to Zika and chikungunya epidemics (data not shown) [27]. Considering the three viruses, the mean incidence rate of probable arboviruscases in the city of Rio de Janeiro in 2016 was 370.80/100,000 [27, 28], 46% lowerthan the incidence in the study area in the same year. In addition, Rio de Janeiro City shows many contrasts. It is composed of both poor communities, such as Manguinhos, and areas with people of high socioeconomic level. Despite being predominantly urban, 29% of its area is covered by vegetation [27]. This heterogeneity of both socioeconomic, climatic and vegetation factors likely explains the lower arbovirus infection rates found in the city as a whole when compared to a specific poor community.

Tropical areas with high population density, informal settlements, low-income level and precarious basic sanitary conditions are those with the highest risk for *Aedes* transmitted diseases [29]. However, spatial analyses studies have shown heterogeneity inside the cities and even within the same neighborhoods are not well explained by these environmental and social indicators. A study on risk factors for dengue in the city of Belo Horizonte, Brazil, showed higher risk areas associated with a lower income of the head of the family, higher household density, and a larger proportion of children and elderly women. Other factors such as basic sanitation, concentration of establishments vulnerable to vector proliferation and population density were not statistically significant in categorizing risk areas [30].

We found a higher rate of arbovirus disease in females. This result should be viewed with caution as it may be the consequence of a higher detection rate in this group since women utilize primary health care services more than men. In addition, Zika has mild symptoms but may cause microcephaly from congenital infections, causing special concern among pregnant women. This further increases the chance of case detection among women.

Although Manguinhos is a subnormal cluster, more commonly referred to as a slum, most residents have access to basic public services like piped water, sewerage system and garbage collection. As a result, these indicators were not useful in determining more deprived areas inside the neighborhood. Nonetheless, the quality of these services is low and there are reports of intermittent water supply in parts of Manguinhos, resulting in the storage of water in unsealed domestic reservoirs [31]. The significance of this issue could not be quantified as there were no data available on the regularity of water supply.

Manguinhos also presents precarious housing in which there are terraces and other hard-to-reach places. As a result, the likelihood of accumulating water increases, which favors the establishment of breeding sites for mosquito larvae. Other problems of the communities are uncovered water boxes, irregularly built community pools, high population density, unsatisfactory garbage collection and the accumulation of building debris in some areas [32–34].

Barcellos et al, 2005, detected that the principal locations with significant potential for dengue transmission were those with many households in a given area, high household density and widespread coverage of water and sewage networks [35]. The present study detected high rates of arbovirus infection in the West and in the North of the community, which are areas of high population density.

In general, people who live in subnormal clusters or slums, like the Manguinhos community, face inadequate living conditions such as low socio-economic status, precarious provision of basic services and informal settlements that have stemmed from unplanned and fast urbanization [36, 37]. Such conditions might create an environment conducive to arbovirus epidemics, and as a result, subnormal clusters have been considered the main contributors to the spread of arbovirus infections in metropolises [36, 38]. In fact, Kikuti et al, 2015 analyzing the risk of dengue disease in a slum in Salvador, observed that lower neighborhood socioeconomic status was independently associated with the increased risk of dengue [29]. However, Teixeira et al, 2002, in a cohort study of 30 neighborhoods in Salvador, did not find a relationship between the incidence of dengue and the standard of living. Neither the seroprevalence nor the incidence of dengue infection was associated with education, income or sex [39].

Teixeira & Cruz, 2011, investigating dengue spatial distribution and socio-environmental indicators in the city of Rio de Janeiro, found a direct association between dengue incidence and social inequality but no association with the social development index [40]. In fact, a systematic review of dengue–poverty relationships found different directions and strengths of this relationship and that there is inconclusive evidence supporting the claim that dengue is a disease of poverty [41]. Researchers investigating the association between dengue incidence and a variety of socioeconomic indicators have produced conflicting results. This may occur because the incidence of these arboviral diseases depends upon the existence of *Aedes aegypti* breeding sites and the immunity of the human population. The presence of these breeding sites is not directly related to poverty but rather depends on diverse characteristics of housing and the organization and use of urban space.

In Manguinhos, we noted a high incidence of arbovirus diseases in areas with relatively better socioeconomic status. This may be a consequence of housing features characteristic of these areas such as uncovered slabs. These slabs are used for diverse purposes and may accumulate water in containers left in the open or even on the floor, where drainage is often imperfect. On the other hand, the houses in the poorest areas do not have slabs or a yard where utensils can be stored in the open, which may explain the lack of association between family income and the risk of arbovirus infection.

According to state public health authorities, serologic tests for chikungunya and dengue should be performed after the fifth day of disease for all suspected cases. However, those who recover in a few days usually do not return to collect serologic tests. Confirmation of Zika reported cases by PCR in the blood and urine was recommended only for pregnant women. In addition, the official reporting of Zika virus illness only became mandatory in Rio de Janeiro in November 2015. As a result, most patients reported as suspected cases did not perform specific laboratory tests, and those who did were tested only for the suspected disease. We found a high positivity rate for chikungunya (73%) and lower rate for Zika (20%). No suspected dengue case was confirmed as dengue.

Since the symptoms of dengue and chikungunya are quite similar in the acute phase of the disease[6], it is likely that most of the cases clinically classified as dengue in the first half of 2015 were in fact Zika and those from 2016 might have been chikungunya or Zika cases. One limitation of our study is that we analyzed data on all reported suspected cases independently of laboratory confirmation. It is possible that other diseases were confounded with these arbovirus infections. Allergic dermatitis could be mistaken for Zika and other unspecific viral diseases could on occasion be confused with any arboviral disease. However, it is important to note that other vector-borne diseases like malaria, yellow fever and typhoid fever are not endemic in the city and there was no rubella transmission either. This lack of precision in the diagnosis may have reduced the strength of the associations found.

As we could not distinguish among dengue, chikungunya and Zika in most cases, we did not focus on exploring each specific disease. Instead, since they are all transmitted by *Aedes* mosquito, we decided to map all reported cases of the three diseases as it could indicate sites with highest vector infestation and factors that favor proliferation and disease occurrence.

Although the present study identified the areas in which there were the highest number of cases, it is not possible to guarantee that individuals were infected near their home. They could have been infected near their job or in other areas of the Manguinhos community. Also, asymptomatic human cases are much more frequent than symptomatic ones, so the number of infected individuals is certainly higher than reported in this study. This inability to detect all infections and to assign the place of infection may have diminished the power of our study in determining high-risk areas.

The results of this study indicate that individuals in vulnerable families are at greater risk of arbovirus infection. The scale used to measure the familiar vulnerability aggregates several details about age, schooling, habitation, employment, drug usage and health conditions. It was not possible to analyze each factor separately as we did not have access to all of the data used to build the scores. Since family income and sanitation indicators were not associated with the risk, some individual characteristics may have contributed to the increased risk. It is possible that individuals with poor health status, as well as the youngest and oldest, are more susceptible to insect bites and symptomatic disease. They are also more prone to seek health care than healthy adults. High population density may have contributed to the risk, as was already reported in other studies.

Several problems must be addressed considering their significant role in the arbovirus infections’ perpetuation. A significant issue is the lack of consistent government efforts and policies to prevent arbovirus infection in Brazil [42]. In general, actions against the proliferation of mosquitoes are taken only during peak months of an epidemic or when there is an outbreak. For example, the United Nations Millennium Development Goals were developed to improve lives and decrease poverty that world leaders approved in September 2000 [43]. The plan to expand the Brazilian sanitary network in 2007 in order to meet the United Nations Millennium Development Goals (Goal 7 –Target 7.C: improve of the access to basic sanitation), which would have reduced the accumulation of still water in domestic reservoirs, was never executed [44].

## Conclusion

Arbovirus infections cause a high burden of disease in Brazilian urban centers. Although we did not distinguish between dengue, Zika and Chikungunya in the cases investigated, our results more broadly explain the risk of diseases transmitted by *Aedes*. Our findings suggest that even in poor neighborhoods, there is a high variability in the risk of acquiring an arbovirus infection. The conditions that favor vector proliferation and infection by arboviruses are complex and involve both individual and environmental characteristics that vary from place to place. In order to reduce the burden of arboviral diseases, continued public health policies and basic services should be provided to the communities at risk that consider specific local needs.
